# The cost-effectiveness of a transdiagnostic intervention to address mental health needs of young people with long-term physical conditions: A commentary

**DOI:** 10.12968/jfch.2025.2.9.392

**Published:** 2025-09-02

**Authors:** Pooja Saini, Lateef Akanni, Zuneera Khurshid, Luís Filipe, Valerio Benedetto

**Affiliations:** Liverpool John Moores University; University of Liverpool; Improvement Academy, Bradford Institute for Health Research; Lancaster University; University of Central Lancashire and NIHR Applied Research Collaboration North West Coast (ARC NWC); Brook Building, University of Central Lancashire, PR1 2HE Preston (UK)

**Keywords:** Children and young people, mental health, long-term physical conditions, cost-effectiveness

## Abstract

Young people with long-term physical conditions are commonly affected by mental health disorders, impacting their quality of life. In this commentary we critically appraised an existing economic evaluation of a transdiagnostic intervention for young people and, based on its findings, we formulated wider implications for practice. While the economic evaluation suitably defined the research question, perspective and time horizon of the analysis, key limitations emerge in relation to the lack of a control group which limits the precision of the cost-effectiveness estimates. Moreover, the design of the transdiagnostic intervention would need further refining as a mix of active and non-active treatments is considered, and their separate contributions to the overall cost-effectiveness could not be ascertained. Despite this, the involvement of staff in lower bands in the transdiagnostic intervention, such as Psychological Wellbeing Practitioners and senior nurses, could be seen favourably from a cost perspective and support its wider implementation.

## Introduction

Long-term physical conditions are common in young people and, while estimates of their prevalence vary in different countries, they constitute an international health burden (Moore et al. 2019). Young people with long-term conditions are at risk of developing mental health disorders three times more than those without conditions (Blackman et al. 2011), with aggravating impacts on their clinical outcomes and overall quality of life (Cottrell 2015).

Compared with young people without mental health problems, the needs of those with long-term conditions affected by mental health disorders trigger a greater use of healthcare resources (Zima et al. 2016) and higher out-of-pocket expenses on the part of the families (Solmi et al. 2018).

To counter the clinical and financial burdens of mental health disorders in this population, there exists evidence suggesting that providing tailored support to treat mental health disorders would have beneficial impacts on physical outcomes (through improved medication adherence for example (Grenard et al. 2011)) and result in long-term savings for the healthcare system (Barnett et al. 2012; Damiano et al. 2023; Kmietowicz 2012). Aligned to this vision is the economic evaluation by Clarke et al. (Clarke et al. 2022) which aimed to determine the cost-effectiveness of a transdiagnostic intervention to address the different mental health needs of young people with long-term physical conditions.

This commentary aims to critically appraise the methods and the results reported in the economic evaluation by Clarke et al. (Clarke et al. 2022) and expand upon the findings in the context of healthcare practice.

## Methods of the economic evaluation by Clarke et al. (2022)

The economic evaluation by Clarke et al. (Clarke et al. 2022) aimed to compare the cost-effectiveness of a brief transdiagnostic intervention in paediatric patients with long-term physical health conditions against standard care. This study was part of the ‘Lucy Project’ single-arm, open, non-randomised trial set at Great Ormond Street Hospital in London. Participants, as part of the intervention, could be allocated to four treatment options: (i) low intensity Cognitive Behavioural Therapy (CBT) delivered by the study team; (ii) neurodevelopmental assessments; (iii) referral to specialist services; or (iv) signposting of resources. No control group was considered. Also, participants could only be included if: (i) they had been a patient at the hospital for a physical health condition in the previous 6 months and were affected by mental health conditions such as anxiety, depression or behavioural problems; or (ii) they were the patient’s caregiver/family member/sibling.

The economic evaluation adopted a healthcare provider perspective (i.e. hospital), with an 18-month time horizon. Costs and clinical outcomes were measured only for the intervention and not for standard care, due to the difficulty in quantifying the related costs. Therefore, costs and outcomes for the comparator were assumed to be equal to zero. Among the costs, resource use data pertained to a variety of direct medical costs, related to staff time and the delivery of low-Intensity CBT, neurodevelopmental assessments as well as resource for signposting, equipment and recruitment materials. Non-medical costs were also included, such as rent and overheads for office spaces and capital costs for the recruitment and treatment booth, computers and administrative items. Resource use data were extracted from the Lucy Project budget which also provided the unit costs for interventions, recruitment materials and equipment. Staff unit costs came from the National Health Service (NHS) pay scales, while overhead and rent unit costs from other publicly available sources. Costs were collected in 2019-2020 and converted into 2020 GBP (using inner London rates).

Outcome data was collected at baseline and 6-months follow-up by using the Paediatric Quality of Life Inventory, with the associated total scores mapped into EQ-5D utilities in order to obtain the quality-adjusted life years (QALYs) used in a cost-utility analyses. QALYs accrued at 6 months were assumed to last for another 6 months, therefore accounting for a whole year. Both costs and outcomes were discounted at a 3.5% annual rate.

Besides a base-case cost-utility analysis centred on the costs and outcomes from the trial, a second cost-utility analysis (i.e. ‘practical’ model) focused on establishing the cost-effectiveness of the intervention by adjusting a few elements. First, the costing figures were streamlined with maximum capacity patient enrolment (assuming that a Psychological Wellbeing Practitioner could see up to 200 patients yearly). Second, the clinical psychologist’s time was doubled and the psychiatrist’s time reduced to reflect their greater and smaller role (respectively) in the allocation of patients to the appropriate treatment. Third, the treatment booth was removed due to patients’ preferences, with cheaper recruitment alternatives adopted. No discount rate was applied in the practical model as all patients were assumed to be seen within one year only (2019).

For both models the authors calculated an incremental cost-effectiveness ratio (ICER) assessed against the UK £20,000-30,000 threshold per QALY gained. Deterministic sensitivity analyses evaluated the impact of changing the values for staff, overhead and capital costs and QALYs (by +/- 20%), the patient counts (up to 200 for the base-case model and down to 93 for the practical model), and the length of the study period (increased to 1.5 years for the practical model) on the resulting ICERs. Probabilistic sensitivity analyses were conducted by attaching plausible distributions to the patient-level outcome and cost data (i.e. normal and beta distributions, respectively) and running 20,000 simulations for both models which were used to create cost-effectiveness acceptability curves.

## Results of the economic evaluation by Clarke et al. (2022)

For the 93 patients who received the intervention in the Lucy Project trial, in the base-case analysis their mean discounted costs were £1,482.09 and their mean discounted QALYs were 0.0698, resulting in an ICER of £21,220 per QALY gained. In the practical model, the mean costs were £309 and the mean QALYs were 0.0709, with the ICER being equal to £4,359 per QALY gained.

In the deterministic sensitivity analyses for the base-case model, increasing the patients count to 200 had the greatest impact on the ICER (53% reduction). Another relevant impact was found when applying a +/- 20% change in the QALYs (reduction in the ICER by 16.5% with a 20% QALYs increase and increase in the ICER by 25% with a 20% QALYs decrease). A similar pattern emerged from running the deterministic sensitivity analyses for the practical model, where the reduction in patients count to 93 brought about the biggest impact on the ICER (115% increase). Significant impacts were also triggered by lengthening the time horizon to 18 months (increase in the ICER by 49%), similar to the base-case model, and varying the QALYs by +/-20% (reduction in the ICER by 17% with a 20% QALYs increase and increase in the ICER by 25% with a 20% QALYs decrease).

The probabilistic sensitivity analyses showed that, both for the base-case and the practical model, 65% of the simulated cost-effectiveness pairs lie in the north-east quadrant, where the intervention produced higher QALYs but higher costs than standard care. The remaining 35% of pairs lie in the north-west quadrant of the cost-effectiveness plane where the intervention would be dominated by standard care. According to the cost-effectiveness acceptability curves, the intervention in the base-case model would have a 49% to 54% likelihood of being cost-effective at the £20,000 to £30,000 per QALY gained threshold; for the practical model, the related likelihood would be 61% to 63%.

## Commentary

### Critical appraisal

We used a mix of questions from existing quality appraisal tools for health economic evaluations (Critical Appraisal Skills Programme 2018; Drummond et al. 2015; Philips et al. 2004) to assess the quality of the study by Clarke et al. (Clarke et al. 2022) (Table 1). Some elements of the study were clearly conducted and reported, such as the definition of the research question, perspective and time horizon of the economic evaluation, and the associated assessment of the clinical outcomes and sensitivity analyses. The interpretation of the results and their generalisability as well as the considerations around the implementation of the intervention were also suitably discussed.

However, other elements present concerns. First, the effectiveness of the transdiagnostic intervention was not based on a systematic review but came solely from the associated Lucy Project trial. While it could be the case that the effectiveness evidence is limited, this should have nevertheless been stated. Second, the absence of a control group (representing standard care) is a key limitation of the study, which is also recognised by the authors. Relatedly, the study lacks a description of what standard care may entail resulting in a somewhat unlikely assumption made about its expected incremental costs and effects (i.e. both assumed to be equal to 0). While it is possible that the transdiagnostic intervention could bring about new costs only compared to standard care, it seems unrealistic to assume that standard care does not generate any effects, i.e. it is effect-neutral. Moreover, it remains unclear how participants in the intervention group were allocated to the four treatment options. While the diversity in the sample in terms of gender, ethnicity and deprivation levels seemed good, sub-group analyses could have also shed some light on which participants accessed the different treatment options, for example by ethnicity, level of deprivation in the area they lived or severity of their long-term physical conditions. More precise results on the sample characteristics for the main trial were also missing as the study presented aggregate figures for the pilot and main study together.

Third, as the authors indicated, the missed inclusion of costs for external services (e.g., for further referrals) could lead to an underestimation of the intervention’s total costs.

### Implications for practice

The findings of the economic evaluation by Clarke et al. (Clarke et al. 2022) highlights a number of potential implications for clinical practice. The transdiagnostic intervention, while showing some promise of cost-effectiveness, needs more precision in terms of what it actually includes. The intervention was presented as a package with four different treatment options, but essentially 3 of them were associated with 99% of the patients’ allocation (i.e. low intensity CBT, referral, and signposting to resources). Moreover, these options represent a mix of active (i.e. low intensity CBT, referral and neurodevelopmental assessment) and non-active treatments (i.e. signposting), rendering difficult to determine the relative contribution to the overall intervention’s cost-effectiveness. The precision of the cost-effectiveness estimation is also put into question by the inclusion in the transdiagnostic intervention of treatment options that could plausibly constitute options offered in standard care (e.g. referral or signposting) (NHS England 2019). Furthermore, any potential combination between two or more of the treatment options was not explored despite being likely in clinical practice (a combination of referral and signposting could be realistic for example (Cantrell et al. 2024)).

Therefore, the lack of precision in the design of the intervention opens questions about its potential implementation, despite the promising cost-effectiveness results. Interestingly, the authors developed a practical model which reflected the economies of scale accrued by reasonably increasing the number of patients seen by a Psychological Wellbeing Practitioner per year. However, any scaling up considerations need to be based on testing the cost-effectiveness of the intervention in multiple sites - as the specificity of the hospital and the connected availability of psychological staff might have played a role in the study - and against a comparator in the control arm.

Despite these uncertainties, there could be also some facilitators to the wider implementation of the transdiagnostic intervention, especially considering that staff in lower bands (with associated lower costs) could be deployed to deliver the intervention, such as Psychological Wellbeing Practitioners (for the low intensity CBT and neurodevelopmental assessment (Green et al. 2014)) and senior nurses (at the triage stage (Stigter-Outshoven et al. 2024)).

### Recommendations for future research

A clear recommendation for future research is associated with the need of running a multi-centre randomised controlled trial to test the cost-effectiveness of the transdiagnostic intervention against standard care. Disentangling the effects of the different treatment options and testing the intervention in multiple regions (beyond London) and settings (beyond secondary care) would also be instrumental in characterising any wider implementation. Future analysis should also break down the results for different sub-groups to check whether the intervention could bring about differential effects and impact on health inequalities. Qualitative feedback from the participants on their perceptions around the intervention would also be beneficial to increase its future acceptability.

## Conclusions

In this commentary we critically appraised the economic evaluation by Clarke et al. (Clarke et al. 2022) on a mental health transdiagnostic intervention for young people with long-term physical conditions. The intervention appeared to be within the UK cost-effectiveness thresholds, and the involvement of Psychological Wellbeing Practitioners and senior nurses added promise to its potential value for money. However, we identified key limitations with the conduct of the analysis which affect the accuracy of the estimates. Importantly, including a control group and refining the intervention in order to understand the relative contribution of the constituting treatments represent gaps which will need to be bridged by future research.

## Figures and Tables

**Table 1:** Critical appraisal tool

*#*	Question	Answer
	**A. Rationale**	
**A1**	**Is there a clear statement of the decision problem?**	⊠ Yes ☐ No ☐ Unclear ☐ Not applicable
	**B. Effectiveness**	
**B1**	**Was the effectiveness of the intervention established on a systematic review?**	☐ Yes ⊠ No ☐ Unclear ☐ Not applicable
	**C. Comparators**	
**C1**	**Was a comprehensive description of the competing alternatives given? (i.e. can you tell who did what to whom, where, and how often)**	☐ Yes ☐ No ⊠ Unclear ☐ Not applicable
	**D. Model perspective and structure**	
**D1**	**Is the perspective of the model clearly stated?**	⊠ Yes ☐ No ☐ Unclear ☐ Not applicable
**D2**	**Are the model structure and its assumptions appropriate and do they fit with the** **clinical theory of the disease process?**	☐ Yes ☐ No ☐ Unclear ⊠ Not applicable
	**E. Costs**	
**E1**	**Were all important and relevant costs for each alternative identified?**	☐ Yes ⊠ No ☐ Unclear ☐ Not applicable
**E2**	**Were costs measured and valued appropriately?**	⊠ Yes ☐ No ☐ Unclear ☐ Not applicable
	**F. Outcomes**	
**F1**	**Were all important and relevant outcomes for each alternative identified?**	⊠ Yes ☐ No ☐ Unclear ☐ Not applicable
**F2**	**Were outcomes measured and valued appropriately?**	⊠ Yes ☐ No ☐ Unclear ☐ Not applicable
	**G. Analysis**	
**G1**	**Was the analysis designed appropriately?**	☐ Yes ⊠ No ☐ Unclear ☐ Not applicable
**G2**	**Were the methods and assumptions used to extrapolate short-term results to final outcomes been documented and justified?**	☐ Yes ☐ No ☐ Unclear ⊠ Not applicable
**G3**	**Was uncertainty in the estimates of costs and outcomes** **adequately characterised?**	⊠ Yes ☐ No ☐ Unclear ☐ Not applicable
	**H. Presentation and discussion of findings**	
**H1**	**Were the results interpreted appropriately?**	⊠ Yes ☐ No ☐ Unclear ☐ Not applicable
**H2**	**Did the study discuss the generalisability of the results to other settings and patient/client groups?**	⊠ Yes ☐ No ☐ Unclear ☐ Not applicable
**H3**	**Did the study allude to, or take account of, other important factors in the choice or decision under consideration (e.g. distribution of costs and consequences, relevant** **ethical issues, or issues of implementation)?**	⊠ Yes ☐ No ☐ Unclear ☐ Not applicable
	**I. Transferability to UK NHS**	
**I1**	**Are the health care system, setting, comparator and patient group comparable to the UK and to the NHS?**	☐ Yes ☐ No ☐ Unclear ⊠ Not applicable

## Key points

- The mental health needs of young people with long-term physical problems worsen their quality of life, but addressing these needs creates costs for the healthcare system and the young people’s families.
- In this study we reviewed a study on the cost-effectiveness of a new transdiagnostic intervention providing mental health care to young people with long-term physical problems.
- The economic analysis was well designed and the involvement of staff in lower bands could save money.
- However, problems exist in the conduct of the analysis as this intervention was not compared against standard care, so it is hard to say if its benefits and costs could be higher or lower than what is provided currently.
- Future research will need to bridge this gap and help refining the value for money of the intervention.

## Reflective questions

- What do you think of the characteristics and overall cost-effectiveness of the transdiagnostic intervention?
- Do you agree with the choice of not including a control group reflecting standard care? Why?
- What barriers and facilitators would you identify to the wider implementation of this transdiagnostic intervention?
